# The Book World of Medicine and Science

**Published:** 1904-04-23

**Authors:** 


### The Book World of Medicine and Science.

The Confessions of a Physician. By V. Veresaeff (V. Smidovich). Translated from the Russian by Simeon Linden. (London: Grant Richards. 1904. Pp. 289. Price 7s. 6d.)

We confess to have derived the greatest of pleasure from reading these confessions. Even if there is an amount of ntrospection which tends at times to become somewhat wearisome, this feeling is to a large extent relieved by the author's evident sincerity and earnestness. For the main part the book is an account of the author's mental attitude towards his environment from the commencement of his studies as a medical student up to the time he is a physician in a fair general practice. To those, therefore, who are interested in the problems of psychology the book will appeal with most force. The author appears to be a very nervous, hyper-sensitive, and conscientious individual, and one, withal, who takes things too seriously to pass by the seeming incongruities of life without putting himself to the task of trying to unravel the tangled skein and analyse the complexities of cause and effect. The development of his views with regard to medicine are quite in accord with what the psychologist would expect. At first he is quite unable to regard the sick patient as a clinical entity; he can only deal mentally with a mass of separate symptoms, often seemingly contradictory. There is no power to group the component parts and the student is lost in bewilderment and totally disheartened. He has the impatience of youth, and because the ablest doctors sometimes disagree and often are mistaken in diagnosis or impotent to cure, his preconceived high opinion of the profession is dashed into pieces. "Side by side with that brilliant medicine of the foot-boards, which heals and resurrects, and for the sake of which I had taken up its study, another medicine slowly revealed itself to me—a helpless, impotent, erring and false science, which undertook the treatment of diseases which it could not identify, painstakingly diagnosed illnesses which it could not cure." As might be expected, a fuller experience allows a juster view, and he little by little comes to wonder that medicine can achieve so much as it does in its strugggle with the infinite intricacies of disease. He learns to appreciate the fact that the eternal fallibility of man is as easy to excuse in a doctor as it is in others. As time goes by the power of forming clinical pictures develops. The multitudinous expressions of disease begin to group themselves in some sort of order in his mind, and the diseased organism is looked upon as a pathological whole, while the perception of the minuter component parts of the picture are more or less relegated by frequent practice to the domain of subliminal consciousness. His mind is ever expanding. Able now to appreciate as one co-ordinate whole the disease, the patient and the medical man's relation to both, his attention is turned to the wider issues involved in the question of disorders as they affect mankind. "One thing was ever becoming clearer and more incontestable in my eyes: that medicine can do no more than point out those conditions which alone make possible a healthy existence and the cure of disease. Therefore, a physician—if he be a physician and not a mummified medical functionary—must first of all strive to remove those conditions which at present render his work both senseless and barren; he must be a public worker in the broadest sense of the term; he must not only prescribe, but he must strive and seek to discover the means for carrying his directions out of theory into actuality." The narrative is enlivened throughout with anecdotes of the author's personal experiences both as a student and practitioner, and there is a good undercurrent of humour, usually at his own expense, in many of these "confessions." With regard to the translation, the book is marred in many places by incorrect spelling and interpretation of medical terms; again, when a Russian journal is mentioned its title has usually been given in the English equivalent, a method which is most confusing, and, we consider, unnecessary. However, these are small matters which do not materially affect the enjoyment which every medical man or student is likely to derive from this excellent narrative, which we are glad to commend to their attention.

A Practical Guide to the Administration of the "Nauheim" Treatment of Chronic Diseases of the Heart in England. By Leslie Thorne Thorne, M.D., B.S. (Durham). Pp. 53, figs. 20, and diagrams 4. (London: Baillière, Tindall and Cox. 1904. Price 2s. 6d. net.)

Much of the subject matter of this modest brochure has already appeared in the *Lancet*. The little work aims at presenting to the practitioner directions regarding the manner and method of employing the so-called "Nauheim" treatment in home life. The action and administration of the baths are discussed and suggestions given regarding the administration of exercises. Much good advice is afforded with respect to the selection of cases, and undoubtedly much wise discernment and sound clinical experience is necessary if the maximum of good is to be obtained with the minimum of risk. Dr. Thorne writes as an enthusiast, but his advocacy is tempered with caution and common sense, and although we cannot consider his figures and diagrams altogether free from the influence of "the personal equation" yet his little work merits critical study and serious attention. Records are given of several cases which have received benefit from the treatment advocated.

Natural Mineral Waters: their Properties and Uses. Eleventh Edition. Revised and Enlarged. Pp. 61. (London: Ingram and Royle, Limited. 1904.)

Natural mineral waters for dietetic as well as therapeutic purposes now find much favour, and in certain circles it is fashionable to undergo "the cure" at the source. This *brochure*, although issued in the interests of the trade, and manifestly partaking of the nature of legitimate advertisement, is nevertheless a most useful little work of reference which physicians interested in climatology, balneology, and hydrotherapeutics will do well to consult. In convenient alphabetical arrangement there are presented many particulars regarding the geographical position, altitude, and character of the various European springs and notes on the action of the waters. We understand a copy of the work may be obtained by any medical practitioner on application to Messrs. Ingram and Royle.

Ambulance Work. Compiled by W. Bagshaw. Revised by H. A. Walker, M.R.C.S., L.S.A., 1903. Pp. 8.

This leaflet consists of the signs and symptoms in insensibility from various causes, arranged in tabular form. Among the nine conditions (exclusive of poisons) with which it deals we note with surprise the omission of uræmia, a form of sudden insensibility of no extreme rarity. We agree with the compiler as to the difficulty ambulance pupils find in distinguishing rapidly between conditions which often bear so close a general resemblance to each other. We would suggest that a future issue might be considerably reduced in size, and got up in a less perishable manner, when it might be carried in the waistcoat pocket for reference.

